# Rapid-onset methimazole-induced lupus in a pediatric patient with Graves’ disease: a case report and review of the literature

**DOI:** 10.3389/fped.2025.1687328

**Published:** 2025-12-08

**Authors:** Nouraldeen Deeb, Salahaldeen Deeb, Mohammad Badawi, Mohammad Alfrookh, Bashar Douden, Saed I. Atawnah

**Affiliations:** 1 Department of Medicine, Al-Quds University, Jerusalem, Palestine; 2 Internal Medicine Department, Al-Ahli Hospital, Hebron, Palestine

**Keywords:** methimazole-induced lupus, Graves’ disease, drug-induced lupus erythematosus, alopecia, case report

## Abstract

Graves’ disease (GD) is the leading cause of autoimmune hyperthyroidism, though pediatric cases are uncommon. Methimazole (MMI) is the first-line therapy; however, drug-induced lupus erythematosus (DILE) secondary to MMI is rare and may be overlooked. We report the case of a 16-year-old girl with newly diagnosed GD who developed inflammatory polyarthritis, a diffuse pruritic maculopapular rash, and alopecia within 1 week of starting MMI (10 mg/day). Laboratory evaluation showed positive antinuclear antibodies and anti-histone antibodies, reduced complement C3 and low-normal C4, and negative anti-double-stranded DNA and extractable nuclear antigen antibodies; urinalysis was normal. MMI was discontinued and oral prednisone (20 mg/day) initiated, resulting in marked improvement within 1 week and complete symptom resolution by days 10–14. This presentation is notable for the unusually rapid onset of MMI-associated DILE in an adolescent and the presence of alopecia, a feature more typical of idiopathic systemic lupus erythematosus (SLE) than classic DILE. The case underscores the importance of maintaining a high index of suspicion for DILE shortly after antithyroid drug initiation, carefully differentiating it from idiopathic SLE using serology and clinical course, and promptly withdrawing the offending agent. Early recognition and short courses of corticosteroids can lead to rapid and complete recovery, while avoiding unnecessary investigations or prolonged morbidity.

## Introduction

Graves’ disease (GD), the principal cause of autoimmune hyperthyroidism, accounts for approximately four in five cases in the United States. Despite a prevalence of nearly 1%, it remains uncommon in pediatric patients with an incidence of 0.1–3 per 100,000 annually. The risk of GD is increased among females, particularly in mid-adolescence, and in those with familial thyroid autoimmunity or coexisting autoimmune disease (1). Antithyroid drugs—most commonly methimazole (MMI) and less frequently propylthiouracil (PTU)—are first-line therapy for GD, with agranulocytosis, hepatotoxicity, and iatrogenic hypothyroidism among the most serious adverse events (1). Drug-induced lupus erythematosus (DILE) constitutes an estimated 5%–10% of systemic lupus erythematosus (SLE) cases and typically emerges after weeks to months of drug exposure. Common features include myalgia, arthralgia/arthritis, cutaneous eruptions, and constitutional symptoms, whereas renal involvement and neurologic involvement are rare (2). In the absence of universally accepted diagnostic criteria, recognition relies on a compatible clinical picture, supportive serology [e.g., positive antinuclear and anti-histone antibodies (AHABs) with low complement in some patients], withdrawal of the culprit drug, and short courses of immunosuppression when indicated (2). Although methimazole has been implicated as a trigger of DILE, pediatric reports are limited and time-to-onset appears variable. We report an adolescent patient with GD who developed inflammatory polyarthritis and a pruritic maculopapular rash within 1 week of initiating methimazole, with serologic support for DILE and rapid resolution after drug withdrawal and corticosteroids, underscoring the potential for unusually early onset and the need for vigilance during antithyroid therapy (1, 2).

## Case presentation

A 16-year-old girl—newly diagnosed with Graves’ disease—presented after 30 days of methimazole therapy (10 mg once daily). She had no prior rheumatologic disease, no drug allergies, no chronic medication use, and had not received immunomodulatory or antibiotic agents before starting methimazole.

Approximately 1 week after methimazole initiation, she developed pain and swelling of the left ankle. Within 2 days, the right wrist, left shoulder, and left middle finger were similarly affected, with mild soft-tissue swelling. She also noted mild alopecia. She self-administered ibuprofen and a short course of amoxicillin–clavulanate with modest relief.

**Figure 1:**
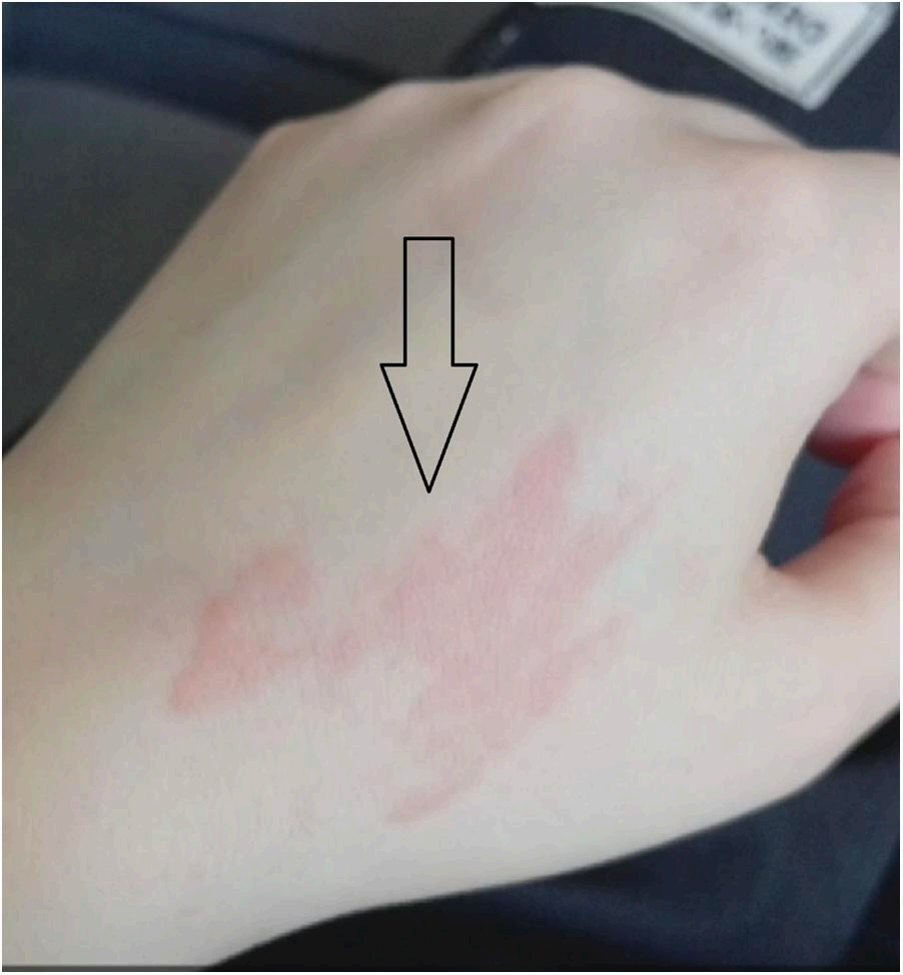
Non-blanching purplish maculopapular rash on the dorsum of the left hand developing after methimazole initiation, resolved following drug withdrawal and corticosteroid therapy.

Two weeks after starting methimazole—about 1 week after the onset of arthralgia—she developed a diffuse, mildly pruritic eruption of erythematous maculopapular lesions on the upper limbs without mucosal involvement or blistering (Figure 1). Given the new polyarthritis and rash, methimazole was discontinued.

Examination showed tenderness with subtle effusions at the left wrist and the left third metacarpophalangeal joint. Thyroid assessment was consistent with Graves’ disease. As part of the systemic work-up for polyarthritis and rash, we sought evidence of extra-articular involvement and alternative etiologies. Chest radiography, spirometry, transthoracic echocardiography, and comprehensive ophthalmologic examination were all unremarkable. Hematologic/biochemical testing showed a normal direct antiglobulin (Coombs) test, an erythrocyte sedimentation rate within the reference range, and normal serum albumin levels. Infectious screening by multiplex viral PCR (including Epstein-Barr virus (EBV), cytomegalovirus (CMV), and parvovirus B19) was negative. Autoimmune serology—including antiphospholipid antibodies (anti-cardiolipin IgG/IgM and anti-β2-glycoprotein I) and extractable nuclear antigen (ENA) panel—was negative; thyroid autoantibodies were not elevated beyond the patient's baseline. Thyroid function tests reflected the underlying thyrotoxicosis under treatment, with no additional systemic abnormalities detected. Urinalysis was normal. Complement levels were reduced (C3 65 mg/dL; reference 90–180 mg/dL) with C4 at the lower limit of normal (10 mg/dL; reference 10–40 mg/dL). Antinuclear antibodies were positive, and anti-histone antibodies were positive, supporting drug-induced lupus (DIL) (Table 1).

**Table 1:** Admission laboratory investigations showing hematologic, immunologic, and thyroid function results at presentation.

Test	Result	Normal range	Normal/ abnormal
Hemoglobin (g/dL)	13.2	12.0–15.5	Normal
WBC count (per µL)	14,000	4,000–11,000	Abnormal
Platelet count (per µL)	210,000	150,000–400,000	Normal
ALT (U/L)	17	7–56	Normal
CRP (mg/L)	11.7	<3.0	Abnormal
Creatinine (mg/dL)	0.8	0.6–1.2	Normal
RF	Negative	Negative	Normal
ANA titer	Positive (1:160)	Negative or ≤1:40	Abnormal
Anti-dsDNA antibodies	Negative	Negative	Normal
ENA profile	Negative	Negative	Normal
Eosinophil count (per µL)	600	0–500	Abnormal
C3 (mg/dL)	65	90–180	Abnormal
C4 (mg/dL)	10	10–40	Low-normal
Anti-histone antibodies (U/mL)	55	0–19	Abnormal

WBC, white blood cell count; ALT, alanine aminotransferase; CRP, C-reactive protein; RF, rheumatoid factor; ANA positive; anti-histone antibodies positive; C3 low with C4 at the lower limit of normal; thyroid profile consistent with hyperthyroidism, supporting drug-induced lupus.

### Diagnostic assessment

Evaluation included thyroid function tests, autoantibody screening, complement levels, and dermatologic assessment of the skin rash. Differential diagnoses such as viral exanthem, juvenile idiopathic arthritis, and idiopathic systemic lupus erythematosus were initially considered. However, the absence of systemic involvement, negative anti-double-stranded DNA (dsDNA) and ENA antibodies, and the temporal relationship to methimazole initiation favored drug-induced lupus. The principal diagnostic challenge was differentiating methimazole-induced lupus from (i) a drug hypersensitivity rash/reactive illness and (ii) *de novo* autoimmune disease. Discontinuation of methimazole and near-simultaneous initiation of prednisone could have accelerated recovery, thereby partially confounding causal inference. Nonetheless, the tight temporal relationship to methimazole initiation, high-titer antinuclear antibody (ANA) with anti-histone antibodies and hypocomplementemia, absence of anti-dsDNA/ENA, lack of renal or neurologic involvement, and sustained remission after withdrawal favored DILE over hypersensitivity or idiopathic SLE.

We considered an intercurrent viral exanthem or a non-specific drug hypersensitivity reaction given the maculopapular rash and low-grade fever. However, the lack of mucosal involvement, no eosinophilia beyond mild elevation, and the presence of high-titer ANA with anti-histone antibodies and low C3 argued against a simple reactive illness. We did not perform a skin biopsy because the eruption was clinically non-specific and improved rapidly after methimazole withdrawal; nevertheless, we acknowledge that histopathology could have further refined the differential.

Prognostically, the favorable course after discontinuation indicated drug causality, although the patient' underlying Graves’ disease diagnosis required ongoing management. She did not meet the 2012 SLICC or 1997 ACR/2019 EULAR–ACR classification criteria for SLE: Mucocutaneous and musculoskeletal items were present, but there was no renal, neurologic, or hematologic cytopenia, or immunologic features beyond ANA/anti-histone antibodies; anti-dsDNA and ENA were negative.

Following methimazole withdrawal, oral prednisone 20 mg daily was initiated and tapered over 3 weeks (20 mg daily for 5 days, then 15 mg for 5 days, 10 mg for 5 days, and 5 mg for 5 days before cessation). Clinical improvement was evident within 7 days of starting prednisone, with complete resolution of rash and arthritis by days 10–14. At the 6-month follow-up, she remained asymptomatic with normalized complement levels (C3 110 mg/dL; C4 22 mg/dL), negative anti-histone antibodies, and a decreased ANA titer of 1:80. At 12 months, she continued to be in remission off corticosteroids; her complement levels were normal, anti-histone antibodies remained negative, and ANA further decreased to 1:40, with no new SLE criteria.

Following methimazole discontinuation, the patient continued to exhibit hyperthyroidism. She was subsequently referred for definitive management of Graves’ disease. Alternative therapeutic options, including propylthiouracil, radioactive iodine ablation, or surgical thyroidectomy, were considered. Given the risk of cross-reactivity with propylthiouracil, the endocrinology team opted to monitor thyroid function closely while planning for definitive therapy.

## Timeline of clinical events

A brief summary of the patient's clinical course and treatment milestones is presented in Table 2.

**Table 2:** Timeline of clinical events and therapeutic interventions in our patient with methimazole-induced lupus.

Day	Event
Day 0	Methimazole (10 mg/day) initiated for newly diagnosed Graves’ disease
Day 7	Onset of joint pain in left ankle
Day 9	Additional joint involvement (wrist, shoulder, finger); self-medicated with ibuprofen and antibiotics
Day 14	Development of diffuse erythematous rash and mild alopecia
Day 15	Methimazole discontinued due to suspected adverse reaction
Day 16	Initiated oral prednisone (20 mg/day)
Day 23	Marked clinical improvement
Day 30	Complete symptom resolution

## Discussion

In this case, SLE developed following the administration of MMI. The patient presented with arthritis, a diffuse erythematous skin rash accompanied by pruritus, alopecia, a positive ANA titer of 1:160, and normal anti-dsDNA and ENA levels. The patient was treated with a low-dose corticosteroid (20 mg/day), resulting in substantial clinical recovery.

Originally described by Hoffmann et al. in 1945, DILE is a rare adverse effect associated with numerous medications, closely mirroring idiopathic SLE (2). Typically, DIL develops after extended drug exposure, though onset can occasionally occur within days or weeks (3). More than 100 agents have been implicated in DIL pathogenesis, including antithyroid drugs like MMI and PTU (4).

MMI-induced lupus is exceptionally uncommon, with only 18 reported cases to date. Its exact pathophysiology remains unclear, but a genetic predisposition may be critical in initiating an aberrant immune response (5). Methimazole is the preferred therapy for adolescent hyperthyroidism, particularly in Graves’ disease, as it targets thyroid peroxidase to inhibit iodine oxidation and prevent the synthesis of thyroxine (T4) and triiodothyronine (T3); however, it does not deactivate circulating hormones (1). Multiple risk factors for DIL have been identified, including female sex, prolonged therapy duration, and higher drug doses (3).

No standardized diagnostic criteria exist for DIL. Common considerations include drug exposure, at least one lupus-like symptom, no previous SLE history, and symptom resolution after discontinuation (3). While a positive ANA test often supports DIL diagnosis, negative ANA does not exclude it if other SLE-related antibodies are detected (3). Clinically, DIL frequently presents with polyarthritis, pruritic rashes, and constitutional symptoms such as fever, fatigue, or myalgia. One study on DILE documented various rashes (urticarial, malar, or vasculitic) in addition to joint and muscle involvement (1, 5). In adolescent cohorts, P-ANCA positivity was found in 40% of cases, whereas ANA positivity reached 80%. Over 90% of DILE patients exhibit anti-histone antibodies, underscoring their significance for diagnostic evaluation and differentiating it from idiopathic SLE (3). Clinical and serological evaluation is crucial for DIL. Studies on childhood-onset lupus also emphasize careful evaluation of thyroid function and associated autoimmune features (6, 7).

Worldwide, 19 cases of methimazole-induced lupus have been documented, including the present case, with only six pediatric cases reported till date (1–5, 8–17), as shown in Table 3. Beernaert and Vanderhulst (2) reported antithyroid drug-induced DIL presenting after months or years of exposure, whereas Seo et al. (9) described MMI-induced DIL within 1 month. In our case, symptoms appeared merely 1 week after MMI initiation, suggesting an unusual rapid onset consistent with MMI-induced lupus, the shortest onset time compared to other cases in the literature.

**Table 3:** Published cases of methimazole-induced lupus: clinical presentation, laboratory findings, treatment, and outcomes.

No.	Year	Age (years)/sex	Clinical presentation	Laboratory results	Time to start symptoms	Treatment	Time to resolution of symptoms	Reference
1		17/F	Fever, arthralgia and erythema, purplish-red erythema	ANA 1:160 (+), anti-dsDNA (+), anti-ssDNA (+)	1 month	Discontinuation of MMI	NR	(8)
2		15/F	Fever, arthritis, rash	LE preparation (+)	3 weeks	Discontinuation of MMI	1 month	(8)
3		14/F	Afebrile, rash, arthritis	ANA 1:160 (+), anti-DNA (−)	18 days	Discontinuation of MMI	72 h	(8)
4		13/F	Afebrile, rash, arthritis	ANA 1: 160 (+), anti-DNA (−)	3 weeks	Discontinuation of MMI	2 months	(8)
5		68/M	Diffuse rash, joint pain	No ANA data	1–2 weeks	Discontinuation of MMI	NR	(8)
6		24/F	Fever, ulcer over legs	ANA 1:2550 (+), anti-dsDNA (+), P-ANCA (+)	4 years	Discontinuation of MMI	2 months	(8)
7		23/F	Fever, generalized rash, migratory polyarthritis, lymphadenopathy	ANA 1:10 (+), anti-dsDNA 123 U/mL	2 weeks	Discontinuation of MMI	10 days	(8)
8		15/F	Bilateral leg edema	ANA 1:640 (+), anti-dsDNA 43.83 IU/mL, MPO-ANCA (+)	2 months	Discontinuation of MMI	40 days	(8)
9	2012	31/F	Rash, multiple bullae	ANA 1:320 (+), AHAB (+), anti-ds DNA (+), ANCA (+).	1 month	Prednisolone, hydroxychloroquine, methotrexate	NR	(9)
10	2002	18/F	Hemoptysis, cough, dyspnea, skin rash	ANA (+) anti-ds DNA (+)	1 week	Methylprednisolone, plasmapheresis	1 month	(10)
11	2016	26/F	Migratory arthritis	ANA 1:320 (+)	1 month	Prednisone	2 months	(13)
12	2024	14/M	Arthralgia, itching	ANA (+), AHAB (−).	2–3 weeks	Discontinuation of MMI	NR	(1)
13	2016	30/F	Skin rash	ANA, ENA, anti-dsDNA, p-ANCA, anti-histones (−)	1 month	Discontinuation of MMI	3 months	(14)
14	2020	32/M	Lymphadenopathy, malar rash, fever, night sweats, arthralgia, Raynaud's phenomenon	ANA 1: 1280 (+), anti-dsDNA (+), anti-nucleosome (+), anti-histone antibodies (+), anti-cardiolipin antibodies (+)	5 months	Hydroxychloroquine, corticosteroids, cyclophosphamide	6 months	(2)
15	2024	41/F	Palpitations, lightheadedness. arthralgia, fever, alopecia, malar rash	ANA: >1:1000 (+), anti-dsDNA (+), AHAB (+)	9 months	Methylprednisolone 1 × 8 mg	3 months	(15)
16	2018	40/F	Fatigue, arthralgia, pruritic rash, aphthous lesions in oral mucosa	ANA 1:640 (+), ANCA 1:160 (+), MPO (+), p-ANCA (+), anti-dsDNA antibodies (+)	11 months	Prednisolone 16 mg/day	6 weeks	(16)
17	1995	24/F	Painful ulcers, fever, pigmented macules	ESR (+), ANA 1: 2560 (+), anti-dsDNA antibodies (+), P-ANCA 1: 320 (+)	4 years	Discontinuation of MMI	2 months	(17)
18	2012	78/M	Shortness of breath, malaise, myalgias, arthralgias, lower limb swelling	RF (+), ANAs 1:640 (+), anti-DNA antibodies (+), ANCA (−)	2 years	Methylprednisolone 500 mg/day, cyclophosphamide 0.5–0.75 g/m^2^	3 weeks	(11)
19 our case	2024	16/F	Arthralgia, and itching, diffuse rash, alopecia, aphthous lesions in oral mucosa, sore throat	RF (−), ANA 1:160 (+), anti-DNA (−), ENA (−), AHAB (+)	1 week	Prednisolone 20 mg/day	1 week	

F, female; M, male; Y, years; ANA, antinuclear antibody; MPO, myeloperoxidase; ENA, extractable nuclear antigen; RF, rheumatoid factor; P-ANCA, perinuclear anti-neutrophil cytoplasmic antibody; AHAB, anti-histone antibodies; LE preparation, lupus erythematosus preparation; anti-dsDNA, anti-double-stranded DNA; anti-ssDNA, anti-single-stranded DNA.

When compared with previously reported cases, our patient exhibited several distinct features. The onset of manifestations within just 10 days of methimazole exposure was among the earliest described in children. Alopecia and a diffuse pruritic rash, which resolved rapidly upon discontinuation of therapy, also add to the spectrum of clinical presentation. Most published pediatric cases reported improvement with drug cessation, occasionally with the addition of corticosteroids or hydroxychloroquine, and variable times to recovery. The concordance of our patient's serologic profile and clinical course with these reports supports the consistency of methimazole-induced lupus as a recognizable clinical entity in pediatrics and highlights the importance of vigilance, particularly in the first few weeks of treatment.

In addition, our report indicates deviations from the standard DIL pattern. Alopecia, often linked to idiopathic SLE but typically absent in DIL, was observed in our patient and only one other case reported by Kusuma et al. (15), mirroring only a handful of MMI-induced lupus cases. Another notable feature was a mildly pruritic, diffuse erythematous rash, which, to our knowledge, has not been previously documented in the literature.

DIL symptoms usually resolve within a few weeks of stopping the offending agent, though some cases can require months (12). Wang et al. (8) observed that a methimazole-induced lupus patient normalized clinically and serologically within 40 days with prednisolone and hydroxychloroquine. In our patient, low-dose corticosteroids brought significant improvement in 1 week, culminating in full recovery within 1 month. This underscores the need for vigilance in patients receiving MMI.

systemic juvenile idiopathic arthritis (sJIA) was included in the differential diagnosis because of the presence fever and rash. However, the clinical pattern was discordant (absence of quotidian fever and evanescent rash; no serositis, lymphadenopathy, or organomegaly). Moreover, prompt remission after stopping methimazole argued against a primary autoinflammatory process.

This case presented diagnostic challenges, given overlapping cutaneous and articular features with drug hypersensitivity and viral/reactive illnesses. The initiation of prednisone contemporaneously with methimazole withdrawal may have contributed to the rapid improvement, potentially confounding attribution. Nevertheless, the serologic pattern (ANA/anti-histone with low C3, negative anti-dsDNA/ENA), lack of end-organ involvement, and durable remission off methimazole support DILE as the most likely diagnosis.

The patient expressed gratitude for the timely diagnosis and treatment, which resulted in rapid symptom resolution and complete recovery. Overall, this case underscores the importance of considering DIL in patients who develop lupus-like symptoms shortly after starting methimazole, especially when presenting with features such as alopecia and pruritic rash, which, although uncommon, may occur.

In cases where methimazole is discontinued due to severe adverse effects such as drug-induced lupus, the long-term management of Graves’ disease must be carefully addressed. Alternative options include propylthiouracil, although cross-reactivity with methimazole has been reported, as well as definitive interventions such as radioactive iodine ablation or thyroidectomy. Our patient has been co-followed by pediatric endocrinology and pediatric rheumatology to guide definitive therapy for Graves’ disease and to monitor for lupus-like relapse.

This case highlights that methimazole-induced lupus, although rare, can present very early and may manifest with symptoms like alopecia and pruritic rash. Recognition of these atypical features, in conjunction with laboratory findings such as positive anti-histone antibodies, is vital for diagnosis. Timely withdrawal of methimazole and corticosteroid administration typically results in full recovery.

This case has several strengths, including detailed documentation of clinical features, serial laboratory evaluation, and a clear temporal relationship illustrated in the patient timeline. It contributes to the very limited pediatric literature on methimazole-induced lupus and emphasizes the need for early recognition of this rare complication. Nevertheless, important limitations remain: The single-patient design, limited duration of follow-up, and absence of confirmatory histopathology constrain generalizability. Causality is inferred primarily from the tight temporal association, the characteristic autoantibody profile, and rapid symptom resolution after drug discontinuation. Even so, these features align with the majority of published reports, supporting the validity of our interpretation and reinforcing consideration of methimazole as a potential cause of lupus-like syndromes in children.

This rare case underscores that methimazole-induced lupus—an infrequent but serious complication of antithyroid therapy—can manifest within 1 week of treatment. Notable features in our patient, including polyarthritis, diffuse erythematous pruritic rash, and alopecia, resolved rapidly after cessation of methimazole and a short course of low-dose corticosteroids. These observations highlight that early recognition and prompt management are essential to prevent severe complications and to optimize patient outcomes.

## Patient perspective

When I first started experiencing joint pain and rashes after taking my thyroid medication, I was very confused and scared. I did n't expect such symptoms to occur so suddenly. I am grateful that the doctors quickly determined the cause and changed my treatment. After stopping the medication and starting the new treatment, I felt better within a week. I am relieved to be healthy again and thankful for the care I received.

## Data Availability

The original contributions presented in the study are included in the article/Supplementary Material; further inquiries can be directed to the corresponding authors.
